# Supplementary material for: Gender favoritism in derogatory and non-derogatory political discourse
Source: PLoS One. 2026 Feb 24;21(2):e0342899. doi: 10.1371/journal.pone.0342899 (PMC12931751; doi:10.1371/journal.pone.0342899)
Supplement: S1 Table — (DOCX) [file pone.0342899.s001.docx]

| **Lexeme** | **Mean pejorative weight** | **Mean political connotation** | **Cluster** |
| --- | --- | --- | --- |
| Aluhut-Träger | 67.40 | 35.77 | L-High |
| Assimilation | 36.02 | 58.29 | R-Low |
| Asylant | 48.12 | 69.25 | R-Low |
| Asylforderer | 51.38 | 73.14 | R-Low |
| Asylwerber | 21.47 | 47.82 | L-Low |
| Atheist | 8.87 | 40.83 | L-Low |
| Ausländerflut | 72.21 | 86.46 | R-High |
| Corona-Diktatur | 67.42 | 81.49 | R-High |
| Coronaleugner | 55.87 | 28.41 | L-High |
| Covidiot | 74.78 | 51.36 | L-High |
| Demagoge | 51.18 | 41.03 | L-High |
| Demokratie | 7.64 | 39.76 | L-Low |
| Diktatur | 51.42 | 55.83 | R-Low |
| Dunkelhäutiger | 27.93 | 43.84 | L-Low |
| Einwanderung | 20.67 | 56.92 | R-Low |
| Emanze | 73.09 | 80.30 | R-High |
| Erinnerungskult | 46.26 | 58.56 | R-Low |
| Erinnerungskultur | 18.35 | 40.56 | L-Low |
| Erstsprache | 9.51 | 44.31 | L-Low |
| Farbiger | 44.45 | 57.20 | R-Low |
| Faschist | 64.66 | 33.06 | L-High |
| Fascho | 67.06 | 25.41 | L-High |
| Feminist | 13.67 | 34.68 | L-Low |
| Flüchtling | 28.71 | 58.04 | R-Low |
| Flüchtlingswelle | 45.11 | 68.74 | R-Low |
| Frauenfeind | 63.89 | 33.02 | L-High |
| Frauenhasser | 68.97 | 35.43 | L-High |
| Geflüchteter | 16.18 | 37.64 | L-Low |
| Gehorsam | 41.93 | 69.26 | R-Low |
| Geldjude | 91.00 | 87.90 | R-High |
| Gender-Irrsinn | 67.99 | 87.76 | R-High |
| Gendern | 16.17 | 32.08 | L-Low |
| geschlechtsspezifische Sprache | 11.82 | 19.59 | L-Low |
| gläubige Person | 7.22 | 40.46 | L-Low |
| Gottloser | 56.67 | 71.17 | R-High |
| Grünfaschist | 71.49 | 86.45 | R-High |
| Grünling | 57.89 | 82.65 | R-High |
| Grünwähler | 36.68 | 69.41 | R-Low |
| Heide | 49.70 | 64.28 | R-Low |
| Heimat | 10.71 | 70.76 | R-Low |
| Heimatland | 17.62 | 77.03 | R-Low |
| Heimatliebe | 21.47 | 79.83 | R-Low |
| Herkunftsland | 15.61 | 55.16 | L-Low |
| Hetzer | 68.01 | 46.81 | L-High |
| homosexuelle Person | 7.51 | 26.59 | L-Low |
| Homosexueller | 12.91 | 39.82 | L-Low |
| Hypermoralismus | 51.78 | 71.05 | R-Low |
| Illegaler | 75.43 | 84.93 | R-High |
| Integration | 9.87 | 38.13 | L-Low |
| Invasion | 59.85 | 76.73 | R-High |
| Invasor | 72.02 | 79.23 | R-High |
| Jude | 21.63 | 60.71 | R-Low |
| Klimaaktivist | 16.52 | 34.30 | L-Low |
| Klimadebatte | 15.28 | 40.23 | L-Low |
| Klimahysterie | 66.66 | 84.15 | R-High |
| Klimakrise | 12.61 | 25.72 | L-Low |
| Klimaterrorist | 74.81 | 85.63 | R-High |
| Klimawahn | 69.27 | 85.01 | R-High |
| Kopftuchfrau | 77.54 | 84.88 | R-High |
| linke Person | 16.03 | 52.10 | L-Low |
| Linker | 29.64 | 68.40 | R-Low |
| Linksfaschist | 71.77 | 86.75 | R-High |
| Mainstream-Medien | 42.80 | 64.07 | R-Low |
| Migrant | 26.92 | 53.82 | R-Low |
| Migration | 10.77 | 46.85 | L-Low |
| Migrationshintergründler | 57.34 | 70.75 | R-High |
| Misogyn | 49.43 | 22.79 | L-High |
| Mohammedaner | 63.63 | 81.02 | R-High |
| Moralismus | 34.22 | 56.60 | R-Low |
| Multikulti | 38.09 | 64.00 | R-Low |
| Multikultiwahn | 71.30 | 86.90 | R-High |
| Muslim | 13.73 | 51.65 | L-Low |
| Muttersprache | 7.16 | 57.31 | L-Low |
| Nationalist | 48.03 | 42.17 | L-High |
| Nationalsozialist | 61.22 | 32.32 | L-High |
| Nazi | 75.25 | 29.46 | L-High |
| Neger | 88.67 | 90.55 | R-High |
| Nigger | 92.80 | 88.69 | R-High |
| Öffentlich-rechtliche | 19.33 | 50.47 | L-Low |
| Pandemie-Management | 15.23 | 42.73 | L-Low |
| Partei | 9.07 | 49.85 | L-Low |
| Patriot | 30.07 | 70.83 | R-Low |
| Person mit Migrationshintergrund | 9.86 | 27.38 | L-Low |
| Person of Color | 8.66 | 13.98 | L-Low |
| Politische Korrektheit | 25.44 | 40.74 | L-Low |
| Pride Month | 6.10 | 17.62 | L-Low |
| Rassist | 63.69 | 27.21 | L-High |
| rechte Person | 30.92 | 28.33 | L-Low |
| Rechter | 46.54 | 24.77 | L-High |
| religiöse Person | 6.47 | 39.41 | L-Low |
| Roma | 30.81 | 49.14 | R-Low |
| Roma und Sinti | 23.60 | 41.96 | L-Low |
| Schuldkult | 65.36 | 80.67 | R-High |
| Schwarzer | 41.91 | 64.79 | R-Low |
| Schwuchtel | 90.36 | 84.89 | R-High |
| Schwuler | 54.46 | 73.48 | R-High |
| Sexist | 59.41 | 29.37 | L-High |
| Sicherheit | 9.97 | 62.35 | L-Low |
| Sozi | 46.94 | 69.61 | R-Low |
| Sozialist | 27.08 | 50.39 | R-Low |
| Sozialleistungsbezieher | 29.86 | 52.17 | R-Low |
| Sozialschmarotzer | 75.74 | 83.73 | R-High |
| Spalter | 62.55 | 50.20 | L-High |
| Stolzmonat | 51.64 | 65.79 | R-Low |
| Südeuropäer | 19.99 | 56.29 | R-Low |
| Südländer | 34.30 | 64.23 | R-Low |
| Systempartei | 60.34 | 64.29 | R-High |
| Transe | 76.52 | 82.03 | R-High |
| Transperson | 8.46 | 27.95 | L-Low |
| Ultralinker | 55.48 | 86.36 | R-High |
| Umvolkung | 75.66 | 87.18 | R-High |
| Vaterland | 27.07 | 84.93 | R-Low |
| Verschwörungstheoretiker | 57.52 | 33.74 | L-High |
| Vielfalt | 8.11 | 23.61 | L-Low |
| Volk | 23.60 | 73.66 | R-Low |
| Volksverdünnung | 77.09 | 83.35 | R-High |
| Waffenbefürworter | 34.57 | 44.49 | R-Low |
| Waffenfanatiker | 54.31 | 30.65 | L-High |
| Widerstand. | 22.02 | 57.00 | R-Low |
| Zigeuner | 78.38 | 82.58 | R-High |
| Zigeunerschnitzel | 59.04 | 76.36 | R-High |
| Zionist | 46.18 | 45.35 | L-High |
