# Supplementary material for: Gender favoritism in derogatory and non-derogatory political discourse
Source: PLoS One. 2026 Feb 24;21(2):e0342899. doi: 10.1371/journal.pone.0342899 (PMC12931751; doi:10.1371/journal.pone.0342899)
Supplement: S2 Table — (DOCX) [file pone.0342899.s002.docx]

| **#** | **Details** | | **Original Wording** | **Deviation Description** | **Reader Impact** |
| --- | --- | --- | --- | --- | --- |
| 1 | Type | Data Preparation | “…categorize the lexemes in terms of political affiliations (left, neutral, right) in a data driven post-hoc manner. For this we will use the 20% most left rated, the 20% most neutrally rated and the 20% most right rated words.” | We conducted k-means clustering utilizing both the political connotation variable and the pejorative weight variable. This approach was motivated by the observation that plotting the two variables against each other revealed that categorizing lexemes solely based on political connotation was insufficient (Fig 2B). The emergence of distinct clusters along the axis of pejorative weight highlighted substantial variation in the behavior of lexemes as a function of differing values of these variables. | Altering the lexeme categorization technique has profound impact on interpreting the results of the current study, since our main analysis procedure is run for each cluster independently. Thus, interpreting group contrasts naturally depends on the underlying lexeme cluster. The complex response pattern regarding the ratings of pejorative weight in women might have remained undisclosed, as differentiating between derogatory and less derogatory word clusters revealed crucial interpretative differences between male and female participants. |
|  | Reason | New knowledge |  |  |  |
|  | Timing | After data access |  |  |  |
| 2 | Type | Analysis | “Secondarily, we conduct separate 2x2 ANOVAs for each word category (left, neutral, right) and each dependent rating variable (political affiliation, pejorative intensity) with participant gender and avatar gender as independent variables.” | We rejected the idea of conducting 2x2 ANOVAs. This was motivated by a presence of non-normality in the data as well as our desire to address within-lexeme and within-subject variation in the model. Consequently, we conducted generalized linear mixed effects models (GLMEMs) for each word cluster (R-Low, L-Low, R-High, L-High) and each response variable (political connotation, pejorative weight) with a random effects structure consisting of the mean Social Dominance Orientation score on a subject basis and a numerically coded word variable. | Using a different statistical model can have large interpretative implications. In this case, results produced by GLMEMs are statistically corrected of the influence of random effects, which is not the case in regular ANOVAs. Furthermore, our GLMEMs partly assume non-normal underlying distributions, as opposed to ANOVAs which require normality of dependent variables. |
|  | Reason | New knowledge |  |  |  |
|  | Timing | After data access |  |  |  |
| 3 | Type | Analysis | “Thirdly, we conduct multiple linear regressions for each word category using political affiliation and pejorative intensity as predictors and social dominance orientation as criterion.” | We opted not to perform multiple linear regressions (MLRs) due to the presence of substantial intercorrelations among predictors within the word clusters. Additionally, conducting these analyses was deemed unnecessary for addressing the primary research questions of the study. | A moderate impact on readers is anticipated. While an entire analytical step was omitted, this pertains solely to a secondary objective of the current study. |
|  | Reason | New knowledge |  |  |  |
|  | Timing | After data access |  |  |  |
