# Supplementary material for: Gender favoritism in derogatory and non-derogatory political discourse
Source: PLoS One. 2026 Feb 24;21(2):e0342899. doi: 10.1371/journal.pone.0342899 (PMC12931751; doi:10.1371/journal.pone.0342899)
Supplement: S3 Table — PG = Participant gender, AG: Avatar gender. *p < .05, ** p < .01, *** p < .001. (DOCX) [file pone.0342899.s003.docx]

| **Domain** | **Cluster** | **Factor** | **Wald *χ^2^*** | ***df*** | ***p*** |
| --- | --- | --- | --- | --- | --- |
| Pejorative weight | R-Low | PG | 1.81 | 1 | 0.179 |
|  |  | AG | 1.77 | 1 | 0.183 |
|  |  | PG x AG | 2.03 | 1 | 0.154 |
|  | L-Low | PG | 0.53 | 1 | 0.465 |
|  |  | AG*** | 12.35 | 1 | 0.000 |
|  |  | PG x AG | 0.98 | 1 | 0.321 |
|  | R-High | PG*** | 90.15 | 1 | 0.000 |
|  |  | AG** | 10.20 | 1 | 0.001 |
|  |  | PG x AG*** | 14.96 | 1 | 0.000 |
|  | L-High | PG*** | 82.10 | 1 | 0.000 |
|  |  | AG*** | 18.71 | 1 | 0.000 |
|  |  | PG x AG*** | 38.30 | 1 | 0.000 |
| Political connotation | R-Low | PG | 0.44 | 1 | 0.509 |
|  |  | AG** | 8.20 | 1 | 0.004 |
|  |  | PG x AG | 1.20 | 1 | 0.272 |
|  | L-Low | PG** | 10.09 | 1 | 0.001 |
|  |  | AG* | 4.21 | 1 | 0.040 |
|  |  | PG x AG | 0.90 | 1 | 0.342 |
|  | R-High | PG*** | 47.35 | 1 | 0.000 |
|  |  | AG*** | 39.71 | 1 | 0.000 |
|  |  | PG x AG*** | 16.75 | 1 | 0.000 |
|  | L-High | PG | 0.83 | 1 | 0.361 |
|  |  | AG* | 6.71 | 1 | 0.010 |
|  |  | PG x AG | 0.33 | 1 | 0.567 |
