# Supplementary material for: Gender favoritism in derogatory and non-derogatory political discourse
Source: PLoS One. 2026 Feb 24;21(2):e0342899. doi: 10.1371/journal.pone.0342899 (PMC12931751; doi:10.1371/journal.pone.0342899)
Supplement: Attachment — Submitted filename: Response to Reviewers.docx [file pone.0342899.s005.docx]

Journal requirements:

1. We checked style requirements again and reformatted our manuscript accordingly.
2. We provided a direct link to a database containing the code and data used in the analyses of the current study.
3. We used an MS Word document for this submission, and we would prefer to continue to work with MS Word.
4. Regarding the financial support statement in the acknowledgements section: Our university explicitly requires us to add this piece of text, acknowledging that the university covers the APCs upon acceptance of the manuscript. As stated, no specific financial support was received for conducting this research.
5. We note that recommended publications should be reviewed before being cited.
6. We double-checked our reference list and made changes where necessary.

Reviewer #1

We would like to thank Reviewer #1 for their comments. We believe their suggestions helped to substantially improve the quality of this work, including but not limited to overall clarity and readability, structural progression, the credibility of sample and item recruitment, and overall methodological soundness.

1. With the intent to promote readability and conciseness, we rephrased the example sentences mentioned by the reviewer. Furthermore, we actively engaged in improving reader comprehension throughout the rest of the manuscript.
2. To clarify in which way our study adds to the current corpus of research, we revised the summary part of our introduction, directly addressing literary gaps this research attempts to fill. (from line 135)
3. To clarify the conceptual and theoretical basis of the two evaluative dimensions used in the current study, pejorative weight and political connotation, we substantially restructured the methods section and introduced an entirely new subsection dedicated to these evaluative measures (see subsection ‘*Pejorative weight and political connotation along a left-right spectrum’ from line 220).*
4. We agree with the reviewer’s views that both the sample and lexeme recruitment passages lacked detailed information. We provided information on the recruitment means and inclusion criteria in the section ‘Participants and anonymity’ (from line 161). Further, we added an entirely new subsection ‘Lexeme pool’ (from line 188) to the methods section, explaining the lexeme curation process in more detail and adding crucial literature references we did not cover in the first version of the manuscript.
5. We agree with the reviewer on that the discussion would benefit from a clearer structure and more streamlined conceptual progression. Thus, we clearly restated our hypothesis and summarized key findings in the beginning of the discussion section, followed by a progression from a broader interpretation of the findings to a more detailed interpretation in the light of RCT and a subsequent turn to SIT. We closed the discussion by providing a summarizing interpretation involving the interactive effects of RCT and SIT. Finally, we informed future work about the implications of the interplay between RCT and SIT and gave recommendations on how to more appropriately disentangle the two.

Reviewer #2:

We would like to thank Reviewer #2 for their valuable comments. We believe their suggestions have significantly improved the quality of this work, enhancing its overall methodological soundness, clarity and comprehensiveness of the limitations and conclusion, as well as the authority of the references.

1. As pointed out by the reviewer, we enriched the sample description by additional characteristics, such as education level and mean SDO expression. Furthermore, we added information on the recruitment means as well as the rationale behind the sample size calculation (from line 161).
2. Limitations: We revised the limitations section (from line 494) of this paper by elaborating on the presentation modality of the lexemes: We elaborated on the shortcoming of exclusively using written lexemes and that future research is advised to not only replicate the mechanisms at play in the current study in a spoken language context, but also to situate political lexemes in a larger discourse context, such as statements or entire speeches.
3. Here we want to provide a response to the last paragraph of the reviewer’s comment:
   a. We agree, to comprehensively draw generalizable conclusions about the presented gender mechanisms, one needs to investigate these in the context of oral and broader context frames. We elaborated on this argument and provided recommendations on how to collectively achieve generalizable results in the limitations section (from line 494).
   b. We acknowledge the notion that our sample size needs to be larger and refer to the newly added part on the rationale of extracting a suitable sample size (from line 166).
   c. We added significant information on the curation of the lexeme pool and provided corresponding literature references. We substantially re-structured the methods section and formed new subsections, one of which dedicated to the lexeme pool itself (from line 188).
   d. To improve the conclusion section (from line 512), we summarized our results in more detail while maintaining a focus on clear language and readability.
   e. The authority of the references has been improved as a consequence of the collective, valuable propositions and comments of all reviewers.

Reviewer #3:

We appreciate Reviewer #3 for their insightful feedback. We are confident that their recommendations have greatly enhanced the quality of this work, particularly in terms of the robustness of the methodology, the validity and scholarly basis of the evaluative measures, the clarity of construct definitions, and the credibility of the references.

1. We elaborated on the literary foundation of our two main evaluative dimensions, pejorative weight and political connotation along a left-right spectrum. We discussed their integration into and comparability with established measures, differential framings and criticism present in the literature, and reported relevant references. Secondly, we discussed the option of using VAS as an evaluative format considering relevant literature. For readability and clearer structural progression, a new subsection ‘*Pejorative weight and political connotation along a left-right spectrum’* was created (from line 220).
2. We agree with the reviewer’s suggestion to report sample characteristics in more detail to provide information the social context in which this study was situated. We extended the ‘Participants and anonymity’ (from line 161) section by recruitment channels as well as educational and political information (represented by the average SDO scores).
3. Lexeme selection: We agree with the reviewer’s views that the curation/selection process needs to be described in more detail as the lexemes represent the core stimuli of the current study. A new subsection called ‘Lexeme pool’ (from line 188) has been added to the methods-section, elaborating on the specifics of the lexeme curation and selection process in more detail, while also adding crucial literature references we did not cover in the first version of the manuscript.
4. Literature regarding lexeme selection: In the course of describing the process of lexeme selection in more detail, we specified relevant literature in the newly added section ‘Lexeme pool’ (from line 188).
